# Activation and Regulation of Indirect Alloresponses in Transplanted Patients With Donor Specific Antibodies and Chronic Rejection

**DOI:** 10.3389/ti.2024.13196

**Published:** 2024-08-20

**Authors:** Sumoyee Basu, Caroline Dudreuilh, Sapna Shah, Alberto Sanchez-Fueyo, Giovanna Lombardi, Anthony Dorling

**Affiliations:** ^1^ Centre for Nephrology, Urology and Transplantation, King’s College London, London, United Kingdom; ^2^ Department of Inflammation Biology, King’s College London, London, United Kingdom; ^3^ Transplantation, Renal and Urology Directorate, Guy’s and St Thomas’ NHS Foundation Trust, Guy’s Hospital, London, United Kingdom; ^4^ Renal Unit, King’s College Hospital NHS Foundation Trust, London, United Kingdom; ^5^ Liver Sciences, King’s College London, London, United Kingdom

**Keywords:** indirect alloresponse, chronic rejection, immune regulation, donor specific antibody (DSA), T follicular helper cells, B lymphocytes

## Abstract

Following transplantation, human CD4+T cells can respond to alloantigen using three distinct pathways. Direct and semi-direct responses are considered potent, but brief, so contribute mostly to acute rejection. Indirect responses are persistent and prolonged, involve B cells as critical antigen presenting cells, and are an absolute requirement for development of donor specific antibody, so more often mediate chronic rejection. Novel *in vitro* techniques have furthered our understanding by mimicking *in vivo* germinal centre processes, including B cell antigen presentation to CD4^+^ T cells and effector cytokine responses following challenge with donor specific peptides. In this review we outline recent data detailing the contribution of CD4^+^ T follicular helper cells and antigen presenting B cells to donor specific antibody formation and antibody mediated rejection. Furthermore, multi-parametric flow cytometry analyses have revealed specific endogenous regulatory T and B subsets each capable of suppressing distinct aspects of the indirect response, including CD4^+^ T cell cytokine production, B cell maturation into plasmablasts and antibody production, and germinal centre maturation. These data underpin novel opportunities to control these aberrant processes either by targeting molecules critical to indirect alloresponses or potentiating suppression via exogenous regulatory cell therapy.

## Introduction

There are three pathways by which transplantation antigens are recognized by CD4^+^ T cells [1–3]. In the “direct” and “semi-direct” pathways, intact donor major histocompatibility complex (MHC) proteins are recognized on the surface of either donor antigen presenting cells (APC) or, in the semi-direct pathway, recipient APC, after MHC transference from donor cells via various routes, including exosome transfer [4]. For detailed description of these pathways, their role and importance in rejection, the reader is referred to several recent reviews [5, 6].

Evidence that a third pathway, called indirect could initiate graft rejection originally came from congenic animal models in which donor and recipient differed only at minor antigenic loci [7–9], and after transplantation of grafts from MHC-deficient rodents [10, 11]. In both, grafts were rejected quickly after activation of self-MHC-restricted CD4+T cells recognising alloantigen presented by recipient APC [12, 13]. The extensive pre-clinical data relating to the role of indirect alloresponses in animal models of transplantation will be briefly reviewed in this introductory section.

**FIGURE 1:**
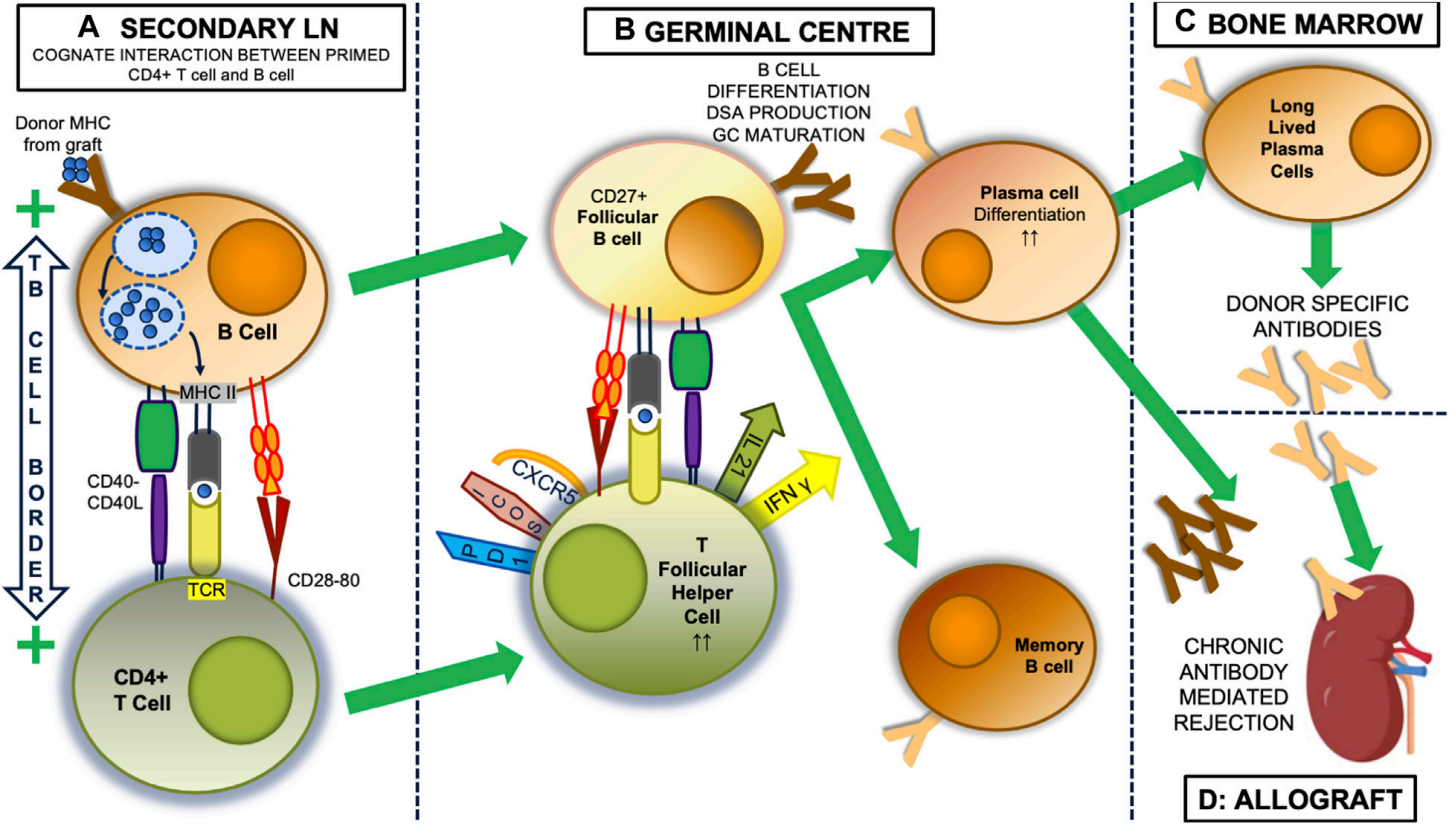
The indirect alloresponse and GC reaction. Within secondary lymph nodes (LN), self MHC-restricted CD4^+^ lymphocytes with indirect allospecificity are primed by dendritic cells that have picked up donor alloantigen, most usually donor proteins encoded by the major histocompatibility complex (MHC), from the allograft and transported it back to the lymph node. (not shown). Once primed, donor specific B cells become the predominant antigen presenting cell **(A)**. These bind donor antigen via their surface immunoglobulin, initially IgM, after which it is internalised and processed into antigenic peptide that then a presented on the B cell surface in the antigen binding groove of MHC class II molecules. At the T: B border in LN, the T cell receptor (TCR) of CD4^+^ T cells can bind this processed peptide, and along with critical interactions between CD40:CD40 ligand, and CD28 and CD80, this interaction activates both the T cell and B cell and the two can enter the germinal centre (GC) response **(B)**. Here, CD27^+^ follicular B cells continue to present antigen to CD4^+^ T cells that have developed a T follicular helper (TFH) phenotype, with expression of CXCR5, ICOS and PD1. Through expression of IL-21 and IFNγ, they drive the production of donor specific antibodies (DSA) initially from plasmablasts, which appear in the circulation and can initiate graft injury, and later from plasma cells, which can be long-lived, after migration to the bone marrow **(C)**. In the process of this happening, the follicular B cells undergo a series of T cell-dependent processes resulting in changes to the structure of their surface immunoglobulin, including isotype and subclass switching (to IgG3), and importantly increasing affinity for alloantigen, which means the DSA also change (in the figure from dark to light brown), being able to bind antigen more avidly and developing ability to activate complement, for instance. These changes are associated with expression of T-bet, IRF4 and Blimp-1 [27]. Memory B cells also emerge from the GC. Thus the unregulated GC reaction results in secretion of DSA with characteristics that can induce acute or chronic rejection in the allograft (D).

Thus, indirectly alloreactive CD4^+^ T lymphocytes exist in the normal repertoire [14, 15], at precursor frequencies lower than T cells activated by direct allorecognition [15, 16], though these frequencies increase after immunisation with soluble MHC [17]. After transplantation, indirectly alloreactive CD4^+^ T cells appear in regional lymph nodes [18, 19], indicating this pathway is activated physiologically. These cells are important, as pre-transplant immunisation with donor MHC causes accelerated rejection [17, 20]. Once activated, indirectly alloreactive CD4^+^ T cells can promote the generation of CD8^+^ cytotoxic T lymphocytes [12], delayed type-hypersensitivity (DTH) responses within the graft [8], and the generation of donor specific antibody (DSA) [8]. DSA are ***only*** generated after indirectly alloreactive CD4^+^ T cells cognately interact with donor-specific B lymphocytes [21–23]. This involves specific differentiation of T follicular helper (TFH) lymphocytes [24] in germinal centres (GC) of secondary lymphoid organs [25, 26] (Figure 1).

Consistent with the crucial role B cells play in the T cell responses to infection [28], there is substantial evidence from animal models that B cells play a central role in indirect alloresponses, especially for the development of chronic rejection (CR). This is likely due to the fact they can undergo clonal expansion through proliferation, and possess specific antigen receptors capable of increasing affinity during an ongoing immune response. For example, when indirect pathway CD4^+^ T cells can only be stimulated by non-B cell APC (such as dendritic or myeloid cells), after mature B cells are prevented from developing [29], or when MHC-deficiency is confined to B cells [30], graft survival is markedly prolonged. In both cases, no DSA develop. In elegant experiments, Zeng et al [31] addressed whether the importance of B cells was to produce DSA, as the prime effector of rejection, or to present antigen to CD4^+^ T cells. They transplanted cardiac allografts into recipients in which B cells were prevented from secreting DSA via the simultaneous knockout of activation-induced deaminase and secretory IgM, but the cells could present antigen. In this model CR lesions developed at the same tempo as in wild-type mice, despite the absence of DSA. The same investigators showed, using MHC-deficient bone marrow chimeras, that self-MHC restricted presentation of allopeptides was needed for CR, as when B cells were present but unable to stimulate the indirect pathway, splenic architecture was maintained but CR was inhibited. These data are consistent with more recent data from Pettigrew’s group, who, working in a similar model nevertheless demonstrated that the ability to generate DSA via a GC response markedly enhanced the speed and severity of the developing CR [26]. Thus, at least in these rodent models, stimulation of CD4^+^ T cells by B cells via the indirect pathway, to generate antibody-independent effector mechanisms, can itself drive the development of CR, but this is significantly enhanced by the presence of class-switched high affinity DSA generated via GC responses. It is being increasingly recognised that the alloantigens that drive indirect allorecognition and maintain B-cell receptor stimulation are transferred to APC via the semidirect-pathway [32].

For the remainder of this review, we will explore clinical data to assess the importance of the indirect pathway for human allograft rejection, particularly CR, review the evidence that this pathway can be suppressed by endogenous regulatory cell populations, and discuss whether this has any potential translational relevance.

## Sensitisation of the Indirect Pathway in Humans Associates With Graft Rejection

Multiple studies have reported an association between pre-transplant donor-specific IFNγ production in enzyme-linked immunosorbent spot assay (ELISPOT) and risk of post-transplant rejection, as analysed in a recent meta-analysis [33]. ELISPOT is a sensitive assay that measures the frequency of cytokine-producing CD4^+^ T cells that are responding to a particular antigen stimulus and IFNγ pathways are significantly upregulated in both biopsy specimens showing rejection, particularly antibody mediated rejection (AMR), and peripheral blood cells of patients with AMR [34], so measuring IFNγ production is logical.

However, most of the studies assessing pre-transplant status have used irradiated whole donor peripheral blood mononuclear cells (PBMC) or splenocytes as the source of donor material, meaning they are likely detecting cytokine production by directly alloreactive lymphocytes as well as CD4+T cells activated by the indirect pathway.

Assays that assess only indirect pathway sensitisation (see Table 1) use donor antigen prepared in ways unable to stimulate direct responses. Saleem et al [35] used synthesised peptides representing donor human leukocyte antigen (HLA) class I to stimulate proliferation of recipient PBMC in 4-day mixed lymphocyte reactions (MLR), and found no responses from 12 kidney transplant recipients (all had had at least 1 episode of rejection) and 3 paediatric heart/lung patients with CR. Iniotaki-Theodoraki et al [36] studied 14 kidney transplant recipients, using APC-depleted donor PBMC in 5-day MLR, and found proliferation in 6 out of 14 patients. Under follow-up, 11 of the 14 maintained stable graft function, 10 of whom showed intermittent indirect alloreactivity. The remaining 3 developed chronic allograft dysfunction (CAD), 2 of whom showed intermittent indirect alloreactivity. Finally, Coelho et al [37] studied 14 living donor kidney transplant recipients using APC-depleted donor PBMC to stimulate proliferation in 9 days MLR. 8 of the 14 showed evidence of indirect alloreactivity, 2 of whom developed CR but in 6, graft function stayed stable. Of the remaining 6 without evidence of indirect alloreactivity, 1 developed CR. The conclusion from these studies, which did not include testing for DSA, was that indirect alloreactivity could be detected in the peripheral blood of long-term renal transplant patients, but this did not seem to predict *future* graft (dys)function. However, none of these studies involved surveillance biopsies.

**TABLE 1:** Summary of functional assays evaluating indirect alloreactive donor specific responses in transplant recipients.

Publication	Study Group	Stimulus	Assay used	Response	Responses after B Cell Depletion	Responses after depletion of CD25hi cells
Saleem et al [35]	12 kidney transplant and 3 paediatric heart lung recipients with history of rejection	Synthesised class I peptides matching donor HLA	Recipient PBMC in 4-day MLR	No responses	N/A	N/A
Iniotaki-Theodoraki et al. [36]	14 kidney transplant recipients	APC-depleted donor PBMC	Recipient PBMC in 5-day MLR	Proliferation^a^ in 6/14 at baseline. Proliferation in 12/14 on serial testing, but not associated with future graft dysfunction	N/A	N/A
Coelho et al [37]	14 kidney transplant recipients	APC-depleted donor PBMC	Recipient PBMC in 9-day MLR	Proliferation^a^ in 8/14. No association with future graft dysfunction	N/A	N/A
Liu et al [38, 39]	32 heart transplant recipients	Synthesised class II peptides matching donor HLA	Serial limiting dilution analyses (detecting proliferating cells) using recipient PBMC *AND* T cells isolated from donor heart	Proliferation^a^ in 18/28 who went onto have episode of rejection within 4 weeks. Correlation between responses from cells in circulation and graft. Association with DSA in patients with CR	NA	NA
Crespo et al [40]	101 kidney transplant recipients	CD2 or CD3-depleted donor PBMC	Recipient PBMC in IFNγ ELISPOT at 3 and 6/12 post-Tx	3-month ELISPOT response^a^ correlated with protocol biopsy-proven rejection at 6 months, and with 24-month DSA development	N/A	N/A
Najafian et al [41]	Recipients of a) HLA-DR-matched kidney transplants (n = 9), HLA-DR mismatched transplants with b) no rejection (n = 11), or c) history of rejection (n = 15)	Synthesised peptides representing hypervariable regions of 5 commonest HLA-DR	Recipient PBMC in IFNγ ELISPOT	Frequency of responding^a^ T cells increased with HLA-DR mismatches and history of rejection	N/A	N/A
Besterd et al [42]	33 kidney transplant recipients	Donor cell membrane preparations	Recipient PBMC in IFNγ ELISPOT	Detectable responses^a^ in 20/33 (60%) – strong correlation with time since Tx and presence of proteinuria	N/A	N/A
Hornick et al [43]	10 heart transplant recipients, 6 with CR. 1 kidney transplant recipient with CR	Donor cell membrane preparations or synthesised donor class I peptides	Limiting dilution analyses (detecting IL-2-producing cells) using recipient PBMC	Detectable responses^a^ in 5/7 with CR but 0/4 without CR	N/A	N/A
Haynes et al [44]	5 cohorts of kidney transplant recipients; a) identical twin donor organ (n = 2), b) clinically tolerant (n = 11), c) stable monotherapy (n = 7), d) standard therapy (n = 18), e) CR (n = 7)	Donor cell membrane preparations, or HLA coated beads	PBMC in trans-vivo assay	Increasing responsiveness^a^ from groups a) – e). Responses reduced in e) with antibodies against IFNγ or IL-17. Responses revealed in a) with antibodies against TGFβ. Responses to HLA coated beads associated with DSA	No impact on responses of two patients	N/A
Vella et al [45]	4 cohorts of kidney transplant recipients; a) HLA-DR MM with CAD (n = 11), b) HLA-DR MM without CAD (n = 10), c) No HLA-DR MM with CAD (n = 5), d) no HLA-DR MM, no CAD (n = 18)	Synthesised peptides representing hypervariable regions of 3 common HLA-DR	Recipient PBMC in 7-day MLR, plus limiting dilution analyses (detecting proliferating cells)	Responses^a^ in 9/11 group a), but 0/10 group b) and 2/23 groups c) and d). Highest frequency of responding cells in group a)	N/A	N/A
Baker et al [46]	22 renal transplant recipients, 9 with CAD	Donor cell membrane preparations	Limiting dilution analyses (detecting IL-2-producing cells) using recipient PBMC	Significantly higher frequencies of responding^a^ cells in the 9 patients with CAD	N/A	N/A
Shiu et al [56, 57]	65 kidney transplant recipients with ‘for cause’ or protocol biopsies 52/65 with AMR	Donor cell membrane preparations	CD8 depleted recipient PBMC in IFNγ ELISPOT	Donor specific IFNy production^a^ in 45/119 (38%). samples. This correlated with reduction in eGFR over time	29/37 (78%) responsive AMR samples had significant reduction in IFNy production compared to 4/8 (50%) in samples from no AMR. In contrast, 17/69 (25%) samples had significant increase in IFNy production	21/66 (32%) samples had significant increase in IFNy production
Shiu et al [58]	51 kidney transplant patients with cAMR.	Donor cell membrane preparations	CD8 depleted recipient PBMC in IFNγ ELISPOT	Donor specific IFNy production^a^ in 58/203 (29%) samples	30/58 (52%) responsive samples had significant reduction in IFNy production	14/30 (46%) samples had significant increase in IFNy production
Burton et al [59]	43 HLA sensitised kidney transplant recipients	PURE HLA proteins matching DSA	CD8 depleted recipient PBMC in IFNγ ELISPOT	Donor specific IFNy production^a^ in 19/98 (19%) samples	13/19 (69%) responsive samples had significant reduction in IFNy production, associated with HLA binding by CD27^+^ B cells. In contrast, 11/98 (11%) samples had significant increase in IFNy production, associated with high proportion of transitional B cells	5/50 (10%) samples had significant increase in IFNy production
Salama et al [68]	23 kidney transplant patients, 8 with previous rejection and CAD.	Donor specific HLA-DR allopeptides	Recipient PBMC in IFNγ ELISPOT	Not reported	N/A	**I**ncreased IFNy production in 6/15 (40%) stable patients but only 1/8 (12.5%) with history of rejection. Responses increased in 8/17 (47%) of all non-responsive samples
Tanaka et al [71]	62 kidney or liver transplant recipients. 17 pre sensitised with DSA	Donor Cells	Recipient PBMC in 5-day MLR	N/A	Significant post-rituximab increase in proliferation by CD4^+^ T cells ONLY in DSA + group	N/A
Schachtner et al [72]	150 blood group compatible (n = 98) or incompatible (n = 52) living donor kidney transplants treated with rituximab induction	Irradiated donor PBMC	Recipient PBMC in IFNγ ELISPOT	Pre-treatment responses^a^ seen in 20/98 (20%) ABO compatible and 12/52 (23%) ABO incompatible patients	Rates of 12-month TCMR were 8/20 (40%) in ABO compatible and 7/12 (57%) in ABO incompatible	N/A

^a^ Detectable responses in all these different assays imply the presence of CD4^+^ T cells that are sensitised to donor antigens.

Abbreviations: AMR, antibody-mediated rejection; APC, antigen presenting cell. CAD, chronic allograft dysfunction; cAMR, chronic AMR; CD8,25,27, cluster of differentiation 8,25,27 +cells; CR, chronic rejection; DSA, donor specific antibody; ELISPOT, enzyme-linked immunosorbent spot assay; HLA- human leukocyte antigen; IFNγ -interferon gamma; IL-2, 17, interleukin-2, 17; MM, mismatch; MLR, mixed lymphocyte reaction; N/A, not applicable; PBMC, peripheral blood mononuclear cells; TCMR–T, cell-mediated rejection; TGFβ, transforming growth factor-beta; Tx–transplant.

In contrast, in 32 heart transplant patients studied within 10 weeks of transplantation, all of whom underwent protocol biopsies, Liu et al [38] isolated PBMC from the circulation and T cells from graft biopsy specimens. They performed limiting dilution analyses to calculate the proportion of recipient CD4^+^ T cells proliferating when stimulated with synthesised peptides representing mismatched donor HLA DR. They detected sensitised T cells in the circulation of 18 of the 28 (64%) patients who went on to have an episode of biopsy proven acute rejection (BPAR) 1–4 weeks later, but only 3/50 samples (6%) when patients had no rejection within the next 1–4 weeks. Moreover, by detecting T cells reacting against the same peptides in the grafts of patients undergoing rejection at up to 10x higher frequencies than in the circulation, they concluded that these T cells play a part in the rejection process. The same group showed, in a separate study [39], that indirect pathway CD4+T cells could be detected in the circulation prior to both episodes of acute and chronic rejection, in the latter case, in association with DSA. Crespo et al [40] performed prospective pre-transplant and 3-month IFNγ ELISPOT analysis in 101 consecutive kidney transplant recipients undergoing a 6-month protocol biopsy. ELISPOT reactivity at 3 months (but not pre-transplant) correlated with sub-clinical BPAR at 6 months, and strongly correlated with DSA development at 24 months. Thus, in contrast to above, these studies involving protocol biopsies in both heart and renal transplant recipients are consistent with the notion that indirect pathway activity is a pre-requisite for both future rejection and DSA development.

Along similar lines, Najafian et al studied indirect alloresponses in several cohorts of renal transplant recipients using recipient PBMC stimulated with synthesised peptides, chosen to represent sequences from the five most frequent donor HLA DR types [41]. They measured ELISPOT IFNγ and found the frequency of cells responding to the allopeptides in healthy controls and DR-matched recipients averaged 4 cells per million CD4+T cells, whereas those in DR mismatched recipients were higher. Frequencies of responding cells were higher still in DR mismatched recipients with a history of rejection, who tended to have frequencies >60 cells per million. Using the same assay, but stimulating PBMC with donor cell membrane antigen preparations, Bestard et al [42] studied 34 renal transplant recipients several years post-transplant, 18 of whom had a history of BPAR. 20 of 33 from whom they had samples had detectable responses to donor antigens, and although there was no correlation between indirect alloreactivity and creatinine there was a strong association with proteinuria and in multivariate analysis, detectable indirect alloreactivity was the only variable associated with proteinuria. These data therefore associate indirect alloreactivity with previous rejection and future graft dysfunction.

Hornick et al [43] found an increased frequency of CD4^+^ T cells capable of producing IL-2 after stimulation with donor membrane antigen preparations in 5 of 7 heart transplant patients with CR but none of the 4 included with no rejection. In kidney transplant patients, Haynes et al [44] used the trans vivo DTH assay to study indirect pathway activation in patients with CR. This assay involves injecting recipient PBMC with donor cell sonicates (as a source of antigen) into the foot pad of an immunodeficient mouse. The degree of swelling that develops over 24 h is proportional to the number of cells responding to antigen. PBMC from patients with CR had the greatest degree of swelling compared to all other patient groups.

Vella et al [45] compared cohorts of kidney transplant patients with and without CAD. This group used synthesised class II allopeptides representing mismatched donor HLA class II to stimulate recipient PBMC in 7-day MLR. Proliferative responses were seen in 9 of 11 patients with CAD but in none of 10 patients without CAD. Responses were seen in only 2 out of 23 controls (for whom the allopeptides did not represent mismatched donor HLA). Proliferative responses were not always seen in serial samples from the same individuals, but it was noted there was evidence of epitope drift in some patients. These authors performed limiting dilution analyses to calculate the proportion of recipient CD4^+^ T cells proliferating, and in CAD, 1 in 9,000 to 1 in 660,000 CD4^+^ cells responded to donor peptides, compared to 1 in 1-2 million cells from controls. Baker et al [46] used a similar approach, using donor cell membrane preparations to stimulate indirect pathway CD4^+^ T cells in 22 renal transplant recipients, 9 of whom had CAD. They showed that the frequency of IL-2 producing cells were significantly higher in the patients with CAD than the patients with stable function. These studies in heart and renal transplant recipients therefore associate T cells primed by the indirect pathway with CR.

Finally, there are numerous studies linking “predicted indirectly recognizable HLA epitopes” (PIRCHE) scores with development of subsequent DSA [47–49] and long-term graft survival [50–53]. As suggested by the name, PIRCHE is an algorithm that identifies parts of mismatched donor HLA that can be presented by recipient HLA class II after processing by APC, so reflects the capacity of specific donor/recipient mismatches to activate the indirect pathway of alloreactivity.

In combination, all these pieces of evidence link sensitisation of CD4^+^ T cells recognising donor antigens via the indirect pathway with previous rejection, and strongly associate the indirect pathway with the development of DSA and subsequent graft dysfunction manifesting as CAD/CR.

## CD4^+^ T Cells With a T Follicular Helper Phenotype Are Involved in Indirect Alloreactivity

Louis et al [54] studied 105 patients, including 20 with DSA and a history of AMR and 31 with DSA but without AMR. In patients with DSA & AMR, there was an over-representation of a CD4 + CXCR5+ TFH subset compared to patients with DSA but no AMR. These cells expressed activation and memory markers and responded to donor cell lysates by expressing IL-21. They could promote DSA appearance when incubated with autologous B cells and their transcriptional profile suggested they were involved in GC responses, providing help to B cells. The DSA in these patients were skewed towards IgG1, IgG3 and C1q binding and the majority of these TFH were Th-1 and Th-17, consistent with a role in isotype switching.

Kenta et al [55] studied indirect alloreactivity in different cohorts of renal transplant recipients, including 12 with DSA pre-transplant, 13 who had developed *de novo* DSA and 33 who were DSA negative throughout. They purified whole CD4^+^ cells or CD45RA+ or CD45RA-negative subfractions (representing naïve and memory cells respectively) and stimulated them with autologous monocytes, differentiated into dendritic cells (DC) *in vitro* and pulsed with donor membrane antigen preparations. These DC mediated emergence of proliferating donor-specific CD4^+^ TFH cells making both IFNγ and IL-21, both key GC cytokines. In the non-sensitised patients, these TFH came mainly from CD45RA+ CD4^+^ cells, as did the IFNγ and IL-21 production, but in DSA+ patients, the CD45RA-negative fraction also contributed.

Both these studies indicate that the CD4^+^ T cells involved in indirect alloresponses adopt a TFH phenotype, capable of secreting IFNγ, IL-17 or IL-21.

## Role and Phenotype of B Cells in Indirect Pathway

Shiu et al [56] used donor membrane antigen preparations to stimulate recipient CD8-depleted PBMC in IFNγ ELISPOTs. In a cohort of 65 patients undergoing protocol or “for cause” biopsy, 52 of whom had AMR, there was evidence of donor specific responsiveness in 38% of samples. Depletion of B cells from the PBMC prior to ELISPOT caused a significant reduction in the frequency of cells producing IFNγ in 29 out of 37 samples (78%) from patients with AMR, but only 4 out of 8 (50%) in samples from controls with non-immune or no pathology on biopsy. Serial changes in patterns of ELISPOT reactivity correlated strongly with changes in eGFR over time [57]. In a second cohort of 51 patients with cAMR studied in a similar way [58], 29% of 203 samples showed evidence of donor specific IFNγ production, 52% of which were dependent on the presence of B cells. Burton et al [59] studied 43 HLA-sensitised patients, using synthesised whole HLA proteins (chosen to match DSA) to stimulate CD8-depleted PBMC. IFNγ production was noted in 23% of 84 samples, 69% of which were B-dependent. In all these studies, IFNγ production was prevented by Btk/Syk inhibitors, leupeptin/pepstatin A/E64-d, or anti-HLA class II blocking antibodies, confirming that alloantigen recognition involved antigen processing and presentation via the indirect pathway. By biotinylating the same HLA proteins, Burton et al were also able to define the phenotype of HLA-binding B cells associated with this pattern of IFNγ production: both class-switched (IgG+) and IgM+ memory (CD27^+^) B cells as well as antigen-experienced (but CD27-negative) marginal-zone precursor B cells appeared to support cytokine production in ELISPOT.

All these data indicate that antigen-experienced, donor antigen specific B cells are present in the circulation of patients with DSA and cAMR and are capable of presenting donor HLA peptides to Th-1, IFNγ secreting CD4^+^ T cells.

Consistent with these data, Louis et al [27] studied B cell phenotypes in the circulation of 96 kidney transplant recipients, 28 of whom had DSA but no AMR, and 20 of whom had DSA with AMR^1^. They found activated memory B cells present in blood and biopsies of patients with AMR. These cells were less frequent in DSA+ AMR- patients. These cells were T bet+, had restricted IGHV gene expression, and were primed for plasma cell differentiation. Importantly, the authors detected DSA secretion after incubation with autologous TFH and the polyclonal activator staphylococcal enterotoxin B, but only from B cells from AMR+ group. These data indicate that there is a difference in the phenotype of donor specific B cells present in DSA+ patients with AMR compared to those with no AMR, with B cells from the former reflecting more GC differentiation than those from the latter.

## Indirect Alloreactivity is Inherently Involved in the GC Reaction

**FIGURE 2:**
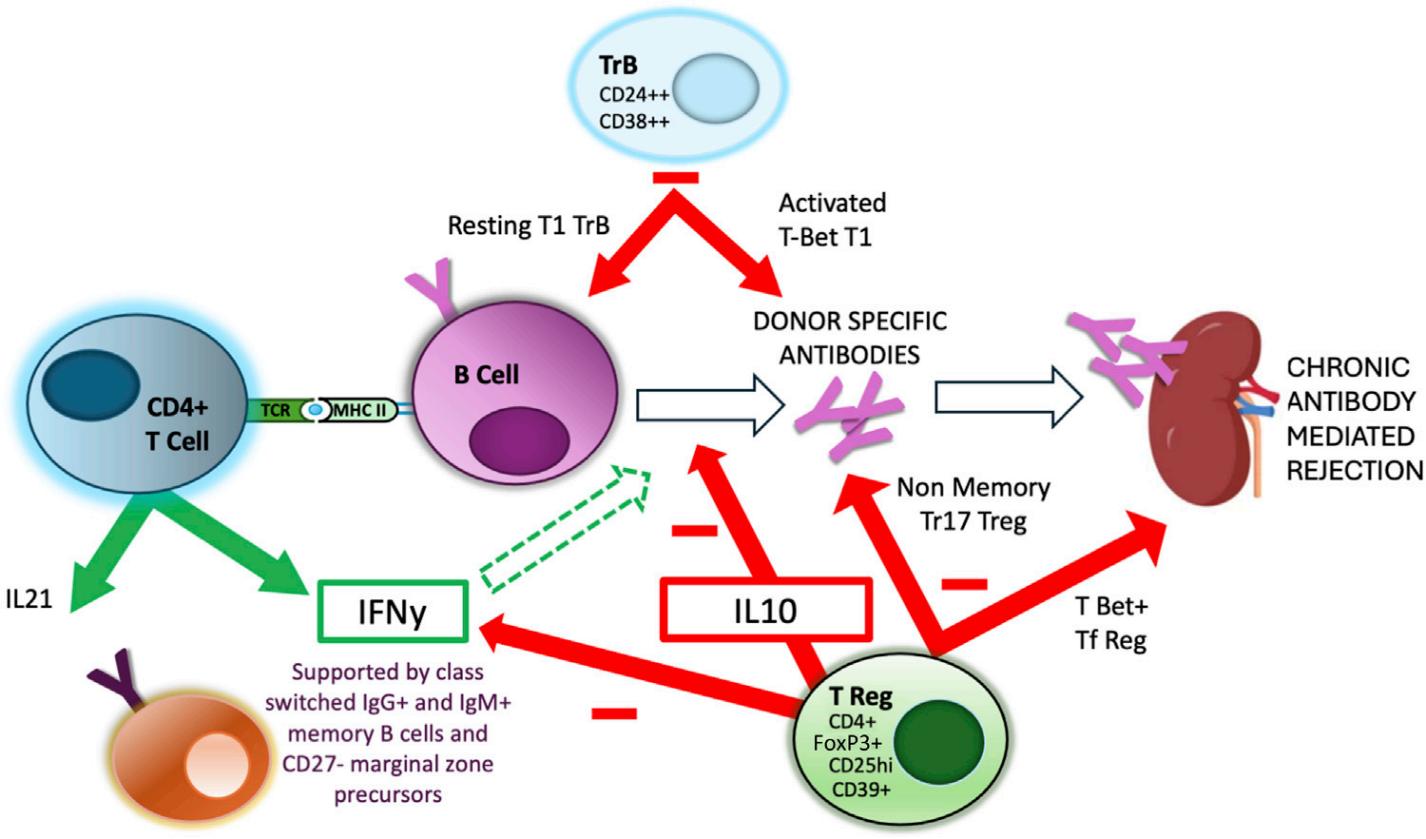
Regulation of Indirect alloreactivity and GC reaction. Aspects of the interaction between CD4^+^ TFH and GC B cells are regulated by specialised regulatory populations of both B cells, expressing high levels of CD24 and CD38 (TrB) and T cells, expressing high levels of CD25, FoxP3 and IL-10. There is evidence that separate and distinct subpopulations of these regulatory cell populations are responsible for regulating different aspects of the GC reaction, including DSA production and GC maturation [67].

GC formation within secondary lymphoid organs during an immune response has been studied extensively within mouse models (Figure 1). Antigen-specific B cells initially undergo clonal expansion within the dark zone of the GC, before moving to the light zone, where continued interaction with TFH recognising the same antigen via the indirect pathway is critical for the GC processes of affinity maturation, driven by somatic hypermutation of immunoglobulin genes, class switching, memory B cell formation and plasma cell differentiation. In humans, it is known that GC TFH cells can be found circulating in the blood [60] and that secretion of IL-21 is a key feature of their GC functionality. Thus there are multiple studies associating the relative proportions of circulating TFH cells post-transplantation with the risk of developing *de novo* DSA [61–63], as discussed by several recent reviews [64–66]. Importantly, many of these GC processes, at least prior to the formation of long-lived plasmas cells, are physiologically regulated as discussed below (Figure 2), representing a potential avenue to therapeutically intervene to prevent DSA development and CAD.

## Physiological Suppression of the Indirect Pathway and Relevance *In Vivo*

### By Tregs

Salama et al [68] were the first to show that depletion of CD25hi regulatory T cells (Tregs) significantly increased indirect IFNγ production in response to donor-specific allopeptides. This was seen in 6 of 15 (40%) stable patients with no history of rejection, but only 1 of 8 (12.5%) of patients with a history of rejection. Of all the non-responsive samples analysed, they found evidence of regulation by Tregs in almost 50%. Interestingly, in a patient in whom serial samples were available, the loss of regulation in the ELISPOT was followed clinically by rejection and graft loss, suggesting that the Tregs were inhibiting anti-donor responses, preventing rejection and that loss of regulation may be a factor in precipitating rejection.

However, Shiu and Burton [56, 58, 59] found that regulation by CD25hi T cells was evident in up to 46% of non-responsive samples from patients with biopsy-proven chronic AMR: in half of these, when Tregs were present, there was complete suppression of IFNγ production and these were the samples that contained the highest proportion of CD4^+^ CD25hi CD39hi Tregs, as assessed by flow cytometry. This suggested that chronic AMR was not associated with a universal loss of the ability to regulate indirect responses. Additional depletion of CD19^+^ cells after CD25hi depletion significantly reduced the frequency of IFNγ+ spots in up to 90% of samples [56], suggesting that Tregs were inhibiting B-cell–dependent indirect alloreactivity; this was seen particularly in samples from patients with AMR. Burton et al went onto show that the phenotype of antigen-binding B cells in these samples were predominantly CD27^−^ naive cells [59], as opposed to the memory phenotypes mentioned above.

Shiu [69] also made interesting observations in a follow-up study of a group of seven highly sensitised kidney transplant recipients, several of whom showed evidence of transplant accommodation [70]. Although 5 of the grafts were lost within 8 years, mostly through CR, two of the accommodated grafts were still functioning 12- and 17-years post-transplantation, both with good transplant function, no proteinuria, but with persisting DSA. ELISPOT analysis showed both had undetectable responses to membrane donor antigen preparations, but responses became evident when CD25hi cells were depleted. Thus, in these highly sensitised individuals with DSA, long term survival associated with complete suppression of indirect anti-donor alloreactivity by Tregs.

### By Bregs

With regard to regulation by B cells, it was also clear from some of the studies above that in some samples, initial depletion of B cells was associated with increases in the frequency of IFNγ producing CD4^+^ cells. This pattern was found in up to 25% of samples [56, 58, 59], and was associated with higher IL-10 production by B cells after polyclonal stimulation [56], lower proportions of HLA binding memory B cells and higher proportions of transitional T1 and T2 B cells (TrB), as assessed by expression of CD38 and CD24 [58, 59]. Importantly, in individuals where serial samples were analysed, two things were apparent. First, both B cell and CD25hi suppression of IFNγ producing cells waxed and waned over time [58]: Second, patients in whom any samples showed IFNγ production in the absence of any regulation appeared to have the worst clinical outcomes [57, 58]. Consistent with this, Haynes et al [44] found no evidence of regulatory B cell activity in the trans vivo DTH assay in patients with CR.

All these data are also consistent with that generated by other groups. Tanaka et al [71] compared pre- and post- rituximab 5-day donor MLRs in 62 kidney or liver transplant recipients, 17 of whom were sensitised with DSA. Pre-rituximab CD4^+^ T cell proliferation, measured using CFSE dilution was equivalent in sensitised and non-sensitised recipients, but in post-rituximab MLRs, there was significantly increased proliferation of the CD4^+^ T cells from the DSA+ group. The authors speculated that B cells were suppressing donor specific CD4^+^ T cells in sensitised recipients.

Consistent with this, Schachtner et al measured IFNγ ELISPOT reactivity in patients receiving either blood group compatible or incompatible kidney transplants [72]. 20% of those receiving ABO compatible kidneys demonstrated pre-transplant anti-donor reactivity, implying prior sensitisation and 40% of these patients had an episode of T cell-mediated rejection in the first year. 23% of those receiving ABO incompatible (ABOi) kidneys had a positive ELISPOT, but the rejection rate in these patients was 57%. Of the various differences between these two populations, one explanation is that rituximab, used exclusively in the ABOi patients, depleted regulatory B cells that spontaneously regulate the indirect alloresponse in some sensitised patients [73], an interpretation consistent with the data generated by Shiu et al [58], who found that rituximab depleted the TrB cells associated with regulation of IFNγ production, but did not deplete the antigen-binding memory B cells responsible for B dependent, non-suppressible IFNγ production.

### Mechanistic Insights

Regarding how these different regulatory populations function, Spadafora-Ferreira et al [74, 75] showed that at least some indirectly alloreactive T cells were FoxP3+ Tregs secreting IL-10. Shiu et al [57] found that in samples with evidence of regulation by B cells, the CD4^+^ T cells making IFNγ were also secreting IL-10, suggesting that regulation involved switching on a well-defined autocrine pathway [76, 77] in Th-1 cells designed to prevent inappropriate IFNγ-driven immunopathology [78]. However, others have defined an important role for B cell-derived IL-10 in regulation of anti-donor alloresponses [79, 80].

Finally, Louis et al, in a second report [67], studied the same patient subgroups (defined above) and found that the numbers and proportions of both Tregs and TrB cells were reduced in DSA+ patients, more so in those who developed AMR. There were significant differences in the qualitative analysis of Treg and TrB cell subsets in the different patient groups. Whereas all DSA+ patients had a deficiency of a non-memory Tr17 Treg subpopulation and a resting T1 TrB subset, only DSA+ AMR+ patients were deficient in both a T-bet+ T follicular regulatory Treg subset and an activated T-bet+ T1 subset of TrB cells. These changes associated with more severe histological features seen in AMR, with the presence of IgG3 and C1q binding DSA and with poorer longer term allograft outcomes. They reported that their regulatory populations limited the APC capacity of B cells, and inhibited TFH proliferation, plasmablast differentiation and IgG secretion. They acted through a combination of direct cell contact (via CTLA4) and IL-10 secretion. The implication of this work was discussed by Basu et al [81]; their data imply separate and distinct tiers of regulation, performed by different subsets of regulatory cells, capable of inhibiting DSA production, but additionally capable of limiting the degree of isotype and subclass switching and affinity maturation within GC. More recently, Dudreuilh et al [82] compared the Treg subpopulations in highly sensitised dialysis patients with those from non-sensitised dialysis patients and healthy controls. Of various differences described, highly sensitised patients had significantly lower proportions of the same two Treg subpopulations identified by Louis et al [67] as being associated with DSA formation and development of AMR, suggesting these deficiencies exist prior to any subsequent transplant.

All these lines of evidence indicate the existence, in patients, of different regulatory populations of T and B cells capable of suppressing mechanisms involved in different aspects of the indirect alloresponse and GC reaction, including donor antigen-specific T cell cytokine production, DSA development and GC differentiation (Figure 2). Importantly, the presence/absence and activity of these populations correlates with graft outcomes. As each layer of regulation may act separately from others, this is one potential reason why the tight links between indirect alloreactivity, DSA formation and clinical phenotype have not always been obvious from the published literature until recently.

## Therapeutic Manipulation of Indirect Alloreactivity

Potential molecular targets to disrupt interactions between TFH and B cells involved in indirect alloimmunity, GC formation and DSA generation have been recently reviewed by Louis et al [65]. An alternative strategy would be to enhance regulatory mechanisms involved in limiting DSA formation or GC maturation. Multiple clinical studies have explored the safety and tolerability of *ex vivo* expanded Treg therapy in transplant patients [66, 83, 84], though most to date have had an emphasis on promoting immunosuppression reduction, minimisation or even elimination in the context of induced immunological tolerance. Second generation studies using manipulated Treg populations, including use of CAR-Tregs, are underway [85].

On the back of the evidence presented above, Dudreuilh et al have initiated a phase 2 clinical trial in sensitised dialysis patients awaiting a transplant, to investigate primarily whether adoptive transfer of ex-vivo expanded Tregs can suppress the CD4^+^ T cell responses to HLA proteins in indirect IFNγ/IL-17 fluorospot assay [86]. Secondary objectives are to determine the proportion of sensitised dialysis patients who may be eligible for a future trial ased on patterns of IFNγ/IL-17A responses to HLA, how long HLA-specific responses remain suppressed, what adverse events associate with Treg therapy, how adoptive Treg therapy changes the number and phenotype of circulating Tregs comparing baseline to post-Treg treatment and finally to determine how adoptive Treg therapy changes HLA Ab profiles. The HLA proteins used are based on the DSA that patients have. The trial is actively recruiting and at the point of writing, has entered the treatment phase, expecting to complete in late 2025. The ambition beyond this is to perform a second trial with clinical endpoints to assess the feasibility of treating highly sensitised patients with Tregs prior to any future transplant. If feasible, this strategy has the potential to improve clinical outcomes in these patients without using significantly enhanced immunotherapy.

## Summary

The indirect alloresponse describes a pathway of antigen recognition involving uptake of donor antigens by APC that are processed into peptides then presented in the antigen-binding grooves of recipient HLA class II to CD4^+^ T cells, following which all immune effector pathways capable of injuring the transplant can be activated. B cells have been shown to be extremely important APC and are necessarily involved in the development of DSA, via a GC reaction and AMR. These B cells have a specific phenotype associated with a GC reaction and can be found in the circulation, particularly in patients with AMR. The indirect pathway also favours the development of CD4^+^ T cells with a TFH phenotype: these can also be found in the circulation, particularly in patients with a history of AMR.

Although initial evidence supported the idea that rejection mediated via the indirect pathway was associated with a loss of immune regulation, newer data support the idea that different aspects of the indirect alloresponse, including CD4^+^ T cell cytokine production, B cell differentiation into antibody secreting plasmablasts, and processes involved in GC maturation, can each be regulated separately by different populations of regulatory T and B cells, including in patients with CR. Importantly the proportion and activity of these populations associates with clinical outcomes. This opens the possibility that CR might be managed by targeting specific molecules involved in the indirect alloresponse, but also the possibility that autologous ex-vivo expanded regulatory populations might be used to treat patients to improve outcomes associated with DSA/AMR without the side effects associated with excessive immunosuppression.

## Footnotes

1 In 17 of the 20, the rejection was classified as mixed AMR/TCMR.
